# Indian Schistosomes: A Need for Further Investigations

**DOI:** 10.1155/2011/250868

**Published:** 2011-10-29

**Authors:** M. C. Agrawal, V. G. Rao

**Affiliations:** ^1^Department of Parasitology, College of Veterinary Science and Animal Husbandry, Jabalpur 482 001, India; ^2^Regional Medical Research Centre for Tribals, Indian Council of Medical Research, Nagpur Road, P.O. Garha, Jabalpur 482 003, India

## Abstract

India is uniquely positioned with regard to schistosomes and schistosomiasis—discovering seven new mammalian species with the existence of three more schistosome species: *Orientobilharzia turkestanicum, O. harinasutai, and Schistosoma haematobium(?)*. An endemic focus of urinary schistosomiasis was reported from Gimvi village of Ratnagiri, Maharashtra with infrequent occurrence of schistosome eggs in human stools. Cercarial dermatitis has been reported to be more abundant in rural population using ponds, tanks, and so forth, for their domestic purposes. Few dermatitis cases were tested positive by CHR. Schistosome antigen was also detected in urine of five cases suggesting existence of active schistosomiasis in India. Nevertheless, human kind does not appear to be the usual host for Indian schistosomes in contrast to *S. haematobium, S. mansoni,* or *S. japonicum*. Various reasons for this phenomenon are discussed including evolution of Indian schistosomes, immune mechanisms, and environmental conditions. These and other aspects such as seasonal effect on the prevalence, snail infectivity, heterologous mating, existence of hybrids, and number of schistosomes in heterologous infections need further investigations with application of molecular techniques. Joint efforts by the national as well as international scientific community would be much more rewarding for better understanding of the parasite and the infection.

## 1. Introduction

India is uniquely positioned with regard to schistosomes and schistosomiasis—discovering seven new schistosome species, namely, *Schistosoma indicum, S. spindalis, S. bomfordi *(Montgomery, 1906), *S. incognitum* (Chandler,1926), *S. nasalis *(Rao, 1933), *S. nairi *(Mudaliar and Ramanujachari, 1945), and *Orientobilharzia dattai* (Dutt and Srivastava, 1955). Additionally, prevalence of *O. turkestanicum* in Srinagar has been confirmed by Dutt and Srivastava [1], while presence of *O. harinasutai* in buffaloes has been suspected long back [2]. This is the country where an endemic area may harbor as many as five schistosome species [3], leading to its own complexities. In contrast to African schistosomes, the life cycle of these blood flukes involves snails inhabiting no big rivers but stagnant water bodies, that is, ponds, tanks, marshy land, agricultural fields, and so forth. Even with presence of nine schistosome species, existence of human schistosomiasis is marred with controversies despite confirming an endemic focus of urinary schistosomiasis (probably by a new schistosome species) in Gimvi village of Ratnagiri district, Maharashtra [4, 5]. There are interesting differences in schistosomes and schistosomiasis of India with regards to conventional schistosomes and schistosomiasis. In this paper, we have attempted to discuss a few features which require attention of international scientific community for better understanding Indian as well as global schistosomiasis.

## 2. Human Schistosomiasis

Schistosomiasis is considered only next to malaria among parasitic diseases of man. Obviously, those schistosome species, namely, *S. haematobium, S. mansoni, S. intercalatum, and S. japonicum, *which cause clinical syndrome in man have attracted almost all the attention of the scientists as well as our scientific organizations, neglecting other schistosome species. In the beginning of twentieth century, India also attracted attention of the scientists on the subject as this was the period of new discoveries on the schistosomes and also because of reporting of several cases of human schistosomiasis from different parts of the country. Soon, it was realized that schistosomiasis cannot establish on the Indian soil as the known intermediaries of human schistosomes are not prevalent; further, trials of infecting local snails with miracidia of *S. haematobium* or *S. japonicum* did not succeed [6]. These findings resulted in fading of the interest of Indian scientists on schistosomiasis. 

Notwithstanding the above facts, a confirmed endemic focus of urinary schistosomiasis was demonstrated in Gimvi village of Ratnagiri district, Maharashtra state of the country [4]. As the eggs were oval shaped and were present in the urine of the patients, the parasite was termed as *Schistosoma haematobium*. But no species of *Bulinus* snail, the intermediate host of *S. haematobium*, is existing in Indian continent; hence a search for its local snail host was extensively made with experimental infection trials with the miracidia. These works resulted in identifying *Ferrissia tenuis* as its intermediate host. Much later, a WHO team, in collaboration with Government of India was able to eliminate the infection from Gimvi village and now it is a dead infection.

However, there are many questions which have remained unanswered in this case [5, 7]. The basic question is about the species of schistosome capable of infecting human beings in Gimvi and also why we have considered it confined only to Gimvi or adjacent villages. The main objection of considering it as *S. haematobium* is that schistosome speciation is still a loose arrangement where egg morphology, snail species, and geographies are taken into consideration while deciding its species [8]. Thus *S. haematobium* is the name given to the African schistosome whose snail host is a *Bulinus* species. Therefore, it will be confusing to call the parasite as *S. haematobium* as neither it is utilizing *Bulinus* species nor existing in known geographies. For this reason, we have suggested to call it a new schistosome species, that is, *Schistosoma gimvicum* [9].

There is no reason to consider this blood-fluke to be confined only to Gimvi village, as *Ferrissia tenuis *snails, so widely prevalent in India [10], were not screened at other places of the country for furcocercous cercariae. It may be emphasized that so far only Gastropods have been incriminated as snail hosts of the schistosomes and no where *Ferrissia *or its related cousin, except in Gimvi village, has ever been incriminated as schistosomes' intermediaries. If *Ferrissia *species is reconfirmed as snail host for any schistosome species from any part of the geography, it will lead to basic changes in our understanding in life cycle of the blood flukes. We have no information if ever comprehensive surveillance of remote areas for urinary schistosomiasis has ever been made in India. This no-reporting does not confirm absence of the infection at other places but may reflect lack of scientific interest in the subject.

It is not that cases of only urinary schistosomiasis were reported from India. Even schistosome infections responsible for hepatic form have been reported from human beings from the country [5]. A confirmed infection of *S. incognitum* was reported from two human cases by Chandler [11] supporting the view of Milton (1919) that a fourth human schistosome is existing in the country [6]. Another support of hepatic infection came forward by reporting schistosome eggs in human stools in Punjab state [12] and also from a village of Andhra Pradesh [13]. But these reports failed to identify new endemic areas of human schistosomiasis, thereby failing to generate interest in the subject by any scientific agency.

The later work on the subject has also not ruled out the probability of occurring human schistosomiasis in India. However, this work was not restricted to examining human stools or urine but was extended to search for cercarial dermatitis. As Indian schistosomes have not developed in line with river system but are using snails inhabiting stagnant waters, the search was made in human beings located near to the ponds and tanks and using them for their routine domestic work including recreational activities. Surprisingly, this surveillance resulted in finding cercarial dermatitis in high proportion in rural/tribal population of Assam [14], Chhattisgarh [15], Madhya Pradesh [16], and even in suburbs of Jabalpur [17].

The cercarial dermatitis may have different sequences as per compatibility of schistosome species involved. In considerable cases and particularly with avian schistosomes, the schistosomulae are destroyed in the skin of man itself. In other cases, at least some schistosomulae are able to traverse to lung or liver where they may be trapped. Still, there is a parasite like *S. japonicum* which causes cercarial dermatitis but able to develop to maturity with laying of eggs in human beings. What is the sequence of cercarial dermatitis in India? It is difficult to predict the same with conformity but positivity of the cases by CHR diagnostic test suggested presence of circulating antibodies hence presence of alive schistosomes in the body of the concerned person [18]. Additionally, five cases turned positive for presence of schistosome antigen in urine when it was tested by a single step diagnostic kit developed by the Netherland laboratory [19]. This prompted us to question if active human schistosomiasis is existing in India [20].

## 3. Schistosome Evolution

Nevertheless, the above bird-view confirms one fact that man is not suffering from schistosomiasis as widely or as acutely by any of the existing Indian schistosomes as is happening with *S. japonicum* or *S. mansoni* or *S. haematobium*. What may be the reasons for this difference? We may get some clue from the hypothesis of Combes [21] who opined that man received schistosome infection by lateral rout and not ventrally. The important argument put by him in support of this hypothesis is absence of any schistosome species in the primates thereby negating its descending to man through primates. The schistosomes have developed either in the ungulates (*S. haematobium* group) or in the rodents (*S. mansoni* group), and it is only in the later part of their evolution when man was incriminated as its host. In line, it may be argued that Indian schistosomes have evolved from original clone and are not descendants of African schistosomes (If we consider origin of *S. indicum* group from African clad of *S. mansoni* or *S. haematobium*, it is difficult to explain exclusion of humans in India as their ancestors, African schistosomes, had developed the ability to infect human beings, by that time), and man escaped from its fury mainly because human population was not concentrated to the infection sites [5]. 

In developing host-parasite relationship, host immune system has played a great role and it might be possible that immune system of Indians is more capable to fight the schistosomes than human beings of other continents. No comparative studies of this type have been made to suggest differences in immunological system of various human populations. Nevertheless, this innate immune system does not appear a simple phenomenon as it does not hold identical for all the schistosome species existing in an endemic area. The best example is of Indian pigs which is resistant to all the schistosome species except *S. incognitum* which thrives well in this host system [22]. Another example may be of sheep which develops three important schistosome species, that is, *S. indicum*, *S. spindale,* and *S. incognitum*. Even the former species has been responsible for out breaks with considerable mortality in the states like Rajasthan [23], Haryana [24], and Southern states [25]. Yet *S. nasale* is found to remain only in the liver of sheep [26], sheep were negative for *S. nasale* in endemic areas [27], or only 2-3% sheep were excreting the eggs in certain areas without any harm [28]. Perhaps, immunological studies of these host species with regards to different schistosome species will be able to throw some light on how host's immune system reacts to different schistosome species it encounters in nature.

## 4. Interactions among Schistosomes

India may prove a correct place to study the interplay among different schistosome species, occurring in same final or intermediate hosts. Here, we will like to cite an example of goats which harbor *S. indicum*, *S. spindale,* and *S. incognitum* at Jabalpur, India. Along with monospecies infection, sympatric infections are also common (this is so in all the ruminants); heterologous infections with *S. indicum* and *S. spindale* or with *S. spindale* and *S. incognitum* are observed but with absence of sympatric infections of *S. incognitum* and *S. indicum *suggesting a strong heterologous immunity between the two [29]. In contrast to the general belief, the absolute number of schistosomes was always high in sympatric infections in comparison to the monospecies infections. How we will explain this contradictory phenomenon? Though *S. spindale* and *S. incognitum* were cohabiting in same goats in nature, our experiments revealed another facet of their survival. In one experiment, seven goat kids were infected simultaneously with equal number of cercariae (2000 each) of *S. spindale* and *S. incognitum* following a new developed polythene-pinna method [29]. The faeces of goats were examined continuously for more than six months; eggs of *S. incognitum* were first to appear and later joined with presence of *S. spindale* eggs. There was not a significant difference in the concentration of the two eggs [30]. However, when the goats were sacrificed at different time intervals, the number of *S. incognitum* flukes was just half to that of *S. spindale*—a situation difficult to assess by egg concentration in the faeces [31]. It is also difficult to explain while female *S. incognitum *possesses only a single egg in its uterus whereas several eggs is the rule in female *S. spindale,* suggesting higher egg laying capacity of the latter. Then why excreting egg numbers of the two species were not differing markedly? At the end of the experiment, when a goat died on 605 DPI, it yielded only male *S. spindale* worms (*n* = 505) with total absence of any *S. incognitum* flukes. This experiment clearly suggested that *S. spindale* was able to knock out all *S. incognitum* flukes from the goats although both parasites got entry in the host at same time, with same number of the cercariae hence with equal opportunity of survival.

Heterologous mating among schistosome species is also not uncommon in the ruminants. We were able to identify mating of *S. indicum* female with male *S. spindale* and vice versa. This heterologous mating could be recognized because of gross morphological differences of the two fluke species [19]. It is difficult to identify heterologous mating between tuberculated blood flukes (*S. indicum* × *S. nasale* × *S. incognitum*) or two atuberculated flukes (*S. spindale* × *Orientobilharzia dattai*) because of difficulty of differentiating the two species in alive condition. The sequel of this heterologous mating has yet not been determined—whether it is able to produce natural hybrids as has been confirmed in African countries.

There are many other observations which need investigations. For example, a seasonal variation in schistosomiasis cases (as determined by faecal examination), when the blood flukes are known to survive more than 2-3 years. A well-known phenomenon is change in prevalence rate of schistosome cercariae emerging from the snails as per season. However, it is worth investigating if reduced number of positive snails in summer season is because of lower number of miracidia available in nature or infectivity of the snails is influenced as per season [32]. 

So far, studies on Indian schistosomes and schistosomiasis have been made using conventional techniques [9]. But further studies on the subject require application of modern techniques like PCR, iso-enzyme, electron microscopy, recombinant techniques, otherwise there are all chances of the studies being remained inconclusive. It will be rewarding if joint studies are made by Indian scientists with international scientific communities and international laboratories working on schistosomiasis. This joint international study is also important since Asia witness existence of two types of schistosome species. On the one hand, we are having Indian schistosomes which are least pathogenic while on the other hand, there is *S. japonicum* which is most pathogenic with widest host rang, including human beings in its spectrum. There is also a need to establish international laboratory in India which is uniquely positioned with regards to schistosomes and schistosomiasis.
